# Pro-Oxidant Anthocyanins-Enriched Fraction Inhibits Androgen Synthesis by Transcriptional Repression of *Cyp17a1* Through *Nr0b2*

**DOI:** 10.3390/antiox15050530

**Published:** 2026-04-23

**Authors:** Giuseppe T. Patanè, Ruben J. Moreira, Ana D. Martins, Pedro F. Oliveira, Stefano Putaggio, Davide Barreca, Marco G. Alves

**Affiliations:** 1 Department of Chemical, Biological, Pharmaceutical and Environmental Sciences, University of Messina, 98166 Messina, Italy; giuseppe.patane@studenti.unime.it (G.T.P.); stefano.putaggio@studenti.unime.it (S.P.); davide.barreca@unime.it (D.B.); 2 Prof. Antonio Imbesi Foundation, University of Messina, 98100 Messina, Italy; 3 Institute of Biomedicine, Department of Medical Sciences (iBiMED), University of Aveiro, 3810-193 Aveiro, Portugal; rubenjesusmoreira@ua.pt; 4 Associated Laboratory for Green Chemistry of the Network of Chemistry and Technology (LAQV-REQUIMTE), Department of Chemistry, University of Aveiro, 3810-193 Aveiro, Portugal; cat.mar@ua.pt (A.D.M.); p.foliveira@ua.pt (P.F.O.)

**Keywords:** Leydig cells, steroidogenesis, anthocyanins, oxidative stress, redox signaling, Cyp17a1, antioxidant paradox

## Abstract

Anthocyanins are plant polyphenols widely regarded as antioxidants, yet they can exert concentration-dependent effects and act as pro-oxidants in specific contexts. Although their protective role in stressed testicular cells is established, their impact on Leydig cell steroidogenesis under non-pathological conditions remains poorly understood. Here, we investigated how an anthocyanin-enriched fraction from *Callistemon citrinus* (0–1.00 μg/mL) affects androgen synthesis in murine TM3 Leydig cells. Cell viability, intracellular ROS, antioxidant capacity, mitochondrial function, androstenedione production, steroidogenic gene expression, and the exometabolome by ^1^H-NMR were assessed. The fraction exhibited biphasic, dose-dependent effects. At 0.01 μg/mL, it induced a mitohormetic response, upregulating mitochondrial complexes III and V. Conversely, higher concentrations (0.10–1.00 μg/mL) reduced metabolic activity, increased intracellular ROS, and significantly suppressed androstenedione synthesis independently of *Star*. These concentrations also induced dose-dependent repression of *Cyp17a1*, concomitant with upregulation of *Nr0b2*, encoding the transcriptional repressor Small Heterodimer Partner (SHP). Overall, the data support a redox-dependent mechanism whereby elevated ROS promotes *Nr0b2* expression, leading to *Cyp17a1* suppression and impaired androstenedione production. These findings challenge the view of anthocyanins as uniformly beneficial for male fertility and identify *Callistemon citrinus* as a sustainable source of bioactive anthocyanins capable of modulating redox–endocrine homeostasis in a dose-dependent manner under basal conditions.

## 1. Introduction

Nowadays, infertility affects 10 to 12% of couples and is defined as the failure to get pregnant after 12 months of unprotected and regular intercourse [1,2]. In this context, male infertility accounts for 30 to 50% of these cases and seems to be increasing at an annual rate of 0.3% [3,4]. Male infertility is a complex disorder triggered by multiple factors, including genetic alterations, oxidative stress (OS), or lifestyle. Nevertheless, unexplained (idiopathic) cases still account for approximately 30–40% of all diagnoses [5]. Hormonal imbalance, particularly altered androgen production, both results from and contributes to fertility issues. In the testis, Leydig cells are primarily responsible for steroidogenesis, crucial for testicular endocrine function, and consequently spermatogenesis. This multi-step process occurs via several cytoplasmic and mitochondrial enzymatic reactions that allow the conversion of cholesterol into testosterone and is regulated by the hypothalamic–pituitary–gonadal (HPG) axis. In these cells, luteinizing hormone (LH) binding activates the adenylate cyclase/cyclic AMP/Protein Kinase A (AC/cAMP/PKA) pathway, increasing the expression of steroidogenic acute regulatory protein (StAR) for cholesterol transport into the mitochondria, and of other enzymes (cytochrome P450 11A1; CYP11A1, CYP17A1, and 17β-hydroxysteroid dehydrogenase type 3; 17β-HSD3) essential for testosterone synthesis pathway [6]. In particular, within the testis, one of the final steps of androgen biosynthesis critically depends on CYP17A1, a key steroidogenic enzyme that catalyzes 17,20-lyase and 17α-hydroxylase reactions, controlling the generation of androgen precursors such as dehydroepiandrosterone and androstenedione in Leydig cells [7]. Given its central position at the branch point of androgen biosynthesis, even moderate changes in CYP17A1 expression or activity can markedly affect systemic androgen levels and male reproductive function. In this context it is also important to elucidate the role of the small heterodimer partner Nr0b2 (SHP), that has emerged as a transcriptional gatekeeper at the crossroads between metabolism and steroid hormone production. In particular, Nr0b2 acts as an inducible corepressor that limits the activity of several nuclear receptors and represses steroidogenic gene expression, thereby exerting a brake on androgen biosynthesis [8]. Previous studies revealed that an impairment of Leydig cell metabolism is correlated with reduced serum testosterone levels and a significant decrease in spermatozoa count [9,10]. Lifestyle habits are among the factors that can affect fertility. For this reason, medical recommendations to improve a couple’s chances include adopting a healthier diet. A Mediterranean diet rich in fruit, vegetables and fish is healthier than the Western diet characterized by high intakes of linoleic acid, fried foods and processed sugars [11,12,13,14]. In fact, the consumption of a Mediterranean diet, characterized by a higher intake of vitamins (A, D, B12, B2), minerals, micronutrients, and α-linoleic acid, is related with an improvement in fertility rates and assisted reproductive technologies (ART) [15]. The consumption of a Mediterranean diet increases omega-3 PUFA derivatives (eicosapentaenoic acid; EPA and docosahexaenoic acid; DHA), crucial for spermatozoa membrane fluidity, maturation, motility, production, and the antioxidant activity of superoxide dismutase 1 (SOD-1) and catalase (CAT), as evidenced by several clinical studies [16,17,18]. In addition, this diet promotes the absorption of polyphenols, a heterogeneous class of natural compounds, that have attracted attention for their antioxidant properties. These compounds may help counteract OS, a condition associated with various diseases, including idiopathic infertility where elevated levels of reactive oxygen species (ROS) have been detected in seminal plasma [19]. However, the presence of ROS is not always negative. OS becomes harmful only when the balance between ROS and antioxidants is disturbed. Regarding male (in)fertility, strong evidence indicates that physiological concentrations of ROS are essential, as they promote key signalling pathways (AC/PKA and RAS/ERK) involved in the acrosome reaction, as well as sperm maturation or membrane fluidity [20,21,22]. However, it remains unclear whether polyphenols exert a truly beneficial effect, and how they act on the various cell types within the testis. Although they share a common structure consisting of three aromatic rings, they can be divided into at least eight subgroups with different properties and biological activities [23]. In this study, we focus on anthocyanins, a subclass of flavonoids, widely present in the Mediterranean diet and known for their strong antioxidant and anti-inflammatory properties [24]. To obtain these compounds, we selected the *Callistemon citrinus* flower (commonly known as the Lemon bottlebrush), a widely distributed ornamental plant in Italy and a sustainable source of anthocyanins. Its use is particularly valuable in the context of green chemistry and in line with the principles of circular economy because it allows the recovery of high yields of anthocyanins as previous reported [25]. Even though several studies highlight how these compounds can improve OS-induced conditions, including aging, inflammation, cardiovascular and neurodegenerative diseases, their impact on testosterone production remains to be elucidated [26,27]. The aim of this work is to evaluate the modulatory effects of an anthocyanin-enriched fraction from *Callistemon citrinus* flowers on steroidogenesis. Using an in vitro Leydig cell model, we aim to unravel how this anthocyanin-enriched fraction affects testosterone production. We further seek to understand if this fraction could be useful as a dietary supplement to mitigate male infertility related to steroidogenesis dysfunction.

## 2. Materials and Methods

### 2.1. Chemicals

Unless otherwise specified, all chemicals and reagents were purchased from Sigma-Aldrich (St. Louis, MO, USA).

### 2.2. Extraction and Preparation of Anthocyanin-Enriched Fraction from Callistemon citrinus

The flowers of *Callistemon citrinus* were harvested in Messina (Sicily, Italy) and used when they reached a moisture < 2%. Specifically, to extract the polyphenolic content and subsequently concentrate the anthocyanins to obtain an anthocyanins-enriched fraction (EX), we followed the green protocol described by Patanè et al. [25]. In particular, after the initial extraction of total polyphenols, anthocyanins were selectively enriched and non-anthocyanin phenolics were largely removed by solid-phase extraction on C18 cartridges. As previously reported by our group, chromatograms recorded at 292 and 330 nm did not reveal relevant peaks attributable to other polyphenol subclasses at levels comparable to anthocyanins [28]. The resulting EX powder was stored in the dark at 4 °C until use. For biological and analytical experiments, fresh stock solutions were prepared in PBS, protected from light, and used within a short time frame to minimize pH- and light-induced degradation of anthocyanins.

### 2.3. Qualitative and Quantitative Analysis of Enriched Fraction in Anthocyanins by RP-HPLC-DAD-ESI-MS/MS

Reverse-phase high-performance liquid chromatography coupled with diode array and electrospray ionization tandem mass spectrometry (RP-HPLC-DAD-ESI-MS/MS) analyses of samples were carried out with a ThermoQuest Model LCQ-Duo equipped with a diode array spectrophotometer and an ion trap mass spectrometer with an electrospray ionization source (ESI) operating in positive ion mode. Chromatographic separation was achieved on a Luna Omega PS C18 analytical column (150 × 2.1 mm, 5 µm) and held in a column oven set at 25 °C. The mobile phase was composed of a liner gradient of solvent A (0.1% formic acid in water) and solvent B (acetonitrile) as follows: 0–3 min, 0% B; 3–9 min, 3% B; 9–24 min, 12% B; 24–30 min, 20% B; 30–33 min, 20% B; 33–43 min, 30% B; 43–63 min, 50% B; 63–66 min, 50% B; 66–76 min, 60% B; 76–81 min, 60% B; 81–86 min, 0% B; 86-90 min 0% B. The total run time was 90 min. The chromatographic conditions were as follows: flow rate, 0.5 mL/min and injection volume of 20.0 µL; and column temperature were 0.4 mL/min. UV–Vis spectral data were collected in the 200–550 nm range, while chromatograms were monitored at 260, 292, 330, 370, and 520 nm to check the eventual presence of different polyphenolic subclasses. Mass spectrometric detection was performed under the following parameters: nitrogen served as the drying gas at a flow rate of 9 L/min and pressure of 40 psi, with a drying temperature of 350 °C. Helium was used as the collision gas (1.46 × 10^−5^ bar) to induce collision-induced dissociation (CID), applying a fragmentation amplitude of 1.0 V in MS/MS mode. Identification was carried out by mean of the comparison of retention times, UV–Vis absorption spectra, and mass spectral fragmentation patterns with those previously described in the literature. Quantification was expressed as cyanidin-3-O-glucoside equivalents per 100.0 g of dry extract (DE), using an external calibration curve prepared with the corresponding reference standard.

### 2.4. Cell Culture—TM3 Mouse Leydig Cells

To study the modulation of natural compounds on steroidogenesis, we use TM3 Mouse Leydig Cells (CRL-1714™) as an in vitro model. This cell line was gently provided by Nafis Rahman, Faculty of Medicine, Institute of Biomedicine, University of Turku (Finland). These are Leydig cells first isolated and characterized from immature male mice at postnatal days 11–13 [29]. We selected this model as these cells produce low concentrations of androstenedione in the absence of hormonal stimulation with LH and human chorionic gonadotropin (hCG) via the AC/cAMP pathway. The cells were cultured in 75 cm^2^ tissue culture flask in 1:1 mixture of Gibco Ham’s F12 medium and Dulbecco’s modified Eagle’s medium (DMEM). The medium was supplemented with 10% (*v*/*v*) fetal bovine serum (FBS) and to avoid contamination penicillin (100.0 U/mL), streptomycin (100.0 µg/mL) and 50.0 µm/mL gentamicin were included in the culture medium. The culture was maintained at a temperature of 37 °C in an atmosphere with 5% CO_2_ and humidity. Every 4 days the culture medium was changed and for the reported experiments were used cells from passages 13 to 25.

### 2.5. Experimental Conditions and Experimental Design

In the initial phase, the cells were kept in culture until 6 biological replicates (n = 6) were obtained to conduct the following experiments. The cells were seeded and exposed for 24 h to increasing concentrations of the anthocyanin-enriched fraction for all experiments, dissolved directly in the media to obtain the following groups: control (CTRL, 0 µg/mL), 0.01 µg/mL, 0.10 µg/mL and 1.00 µg/mL. The concentrations were selected based on literature data, as they correspond to low, moderate, and high anthocyanin exposure levels achievable through dietary intake or nutraceutical supplementation [30,31]. Experiments of cytotoxicity, metabolic activity, intracellular ROS quantification and mitochondrial membrane potential (ΔΨm) analysis were performed. The exposures were carried out on 100 mm Petri dishes in the presence of hCG (3.0 ng/mL) to stimulate steroidogenesis and with the anthocyanin-enriched fraction also for 24 h. Following exposure, the medium was collected to assess total antioxidant potential (Ferric reducing antioxidant power—FRAP assay), total androstenedione production (ELISA) and exometabolome (^1^H-NMR). The TM3 cells were trypsinized and counted. DNA, RNA and proteins were isolated from the cells to evaluate gene expression of steroidogenesis and mitochondrial function markers via RT-qPCR, and to quantify oxidative phosphorylation complexes and oxidative stress via Western/slot blot.

### 2.6. Evaluation of Metabolic Activity and Cytotoxic Profile

#### 2.6.1. Metabolic Activity

To perform the assay, cells were seeded in a 48-well plate at a density of 30,000 cells/well and allowed to grow to 70% confluence in DMEM: Ham’s F12 medium. After the 24 h exposure, the culture medium was aspirated, and the cells were washed with 500.0 μL of PBS and then fresh serum-free medium and MTT solution (5.0 mg/mL, solubilised in PBS) was added, as reported by Martins and collaborators [32]. The plate was incubated for 2 h at 37 °C and protected from light. After incubation, the medium was aspirated and 250.0 μL of DMSO was added to facilitate the solubilization of the formazan crystals obtained. Then 100.0 μL from each well was transferred to a 96-well plate and the blank was prepared with DMSO, and readings were taken at 570 and 655 nm with multiplate reader (MultiSkan Go, Thermo Fisher Scientific, Vantaa, Finland). To perform the statistical analysis, after subtracting the respective blanks from each data, the absorbance at 655 nm was subtracted from the value obtained at 570 nm. All exposures were performed in six independent biological replicates (n = 6), and each experimental condition was analyzed in duplicate.

#### 2.6.2. Lactate Dehydrogenase (LDH) Activity

The experiment was performed following the guidelines of the LDH Cytox^TM^ Assay Kit (BioLegend^®^, San Diego, CA, USA). In detail, cells were seeded in their culture medium in a 48-well plate at a density of 30,000 cells/well. After the exposures, 100.0 μL were taken from each well in duplicate and transferred to a 96-well plate for analysis. 100.0 μL of LDH assay buffer was added to each well and allowed to incubate in the dark for 30 min at 37 °C, as suggested by the manufacturer. At the end of the incubation, 50.0 μL of stopping solution was added and the LDH release analysis was done at 490 nm using a multiplate reader (MultiSkan Go, Thermo Fisher Scientific, Vantaa, Finland). All analyses were performed as described above (n = 6).

#### 2.6.3. Proliferative Activity

Cell viability and proliferation were analyzed by SRB colorimetric assay. In detail, the cells were seeded in a 48-well plate at a density of 30,000 cells/well to obtain 60% confluence (n = 6). After 24 h exposures, the medium was removed, and the cells were washed twice with PBS at room temperature. Fixation solution (1% Acetic acid solution in 99% methanol) was added and left at −20 °C for at least 1H. After fixation, the solution was removed from each well and the plate was left open at a temperature of 37–40 °C to facilitate drying. The cells were then exposed to 300.0 μL of 0.05% SRB solution in 1% acetic acid in water for 1 h. Finally, the dye solution was removed, and each well was washed carefully with 1% acetic acid in water to remove all SRB solution not bound to the cell protein from the wells. After drying the plate, 250.0 μL of 10.0 mM TRIS solution (pH 10) was added to solubilise the cell protein-bound probe. For signal interpretation, 100.0 μL of each experimental condition was transferred in duplicate to a 96-well plate, and absorbance was measured at 490 nm using a multiplate reader (Multiskan GO, Thermo Fisher Scientific, Vantaa, Finland). A 10.0 mM TRIS solution (pH 10) was used as a blank.

### 2.7. Detection of Total Intracellular Reactive Oxygen Species (ROS)

Intracellular ROS production was detected using CM-H_2_DCFDA (C6827, Invitrogen™, Carlsbad, CA, USA), which is a chloromethyl derivative of H_2_DCFDA, and HOECHST (H3570, ThermoFischer Scientific, Waltham, MA, USA) for normalization of results, as reported by Gomes-Andrade and collaborators [33]. Cells were seeded in a black fluorescence plate as described (n = 6) and allowed to grow to a confluence around 70-80%. After exposure, the wells were washed with PBS and incubated for 30 min at 37 °C in the dark with 100.0 μL of a solution containing CM-H_2_DCFDA (5.0 μM in PBS) to detect total ROS and HOECHST (1.0 μL/mL) for normalization of results. To quantify ROS and cell number, fluorescence was read at 495/529 nm (Ex/Em) and 350/461 nm (Ex/Em) respectively, with a Synergy™ H1 multi-mode microplate reader (BioTek, Winooski, VT, USA). Finally, the results were normalized for cell nuclei content and expressed in DCF Fluorescence Units. To acquire representative images of intracellular ROS, TM3 cells were seeded on Ibidi μ-slide 8-well plates (Ibidi GmbH, Grafelfing, Germany) at a density of 3000 cells/well, incubated overnight to allow cell adherence, and exposed to the experimental conditions. After 24 h, the cells were incubated with the CM-H_2_DCFDA/HOECHST solution previously described and observed using a Zeiss LSM 880 Airyscan confocal microscope (Zeiss, Oberkochen, Germany), equipped with a 63×/1.4 NA Plan-Apochromat oil-immersion objective. Image acquisition was controlled by the ZEN software 2.3 (Zeiss), using appropriate laser lines and filter settings for HOECHST and CM-H_2_DCFDA.

### 2.8. Mitochondrial Membrane Potential (*Δ*Ψm)

The ΔΨm of Leydig cells was detected by using 5,5,6,6′-tetrachloro-1,1′,3,3,3′ tetraethylbenzyme-dazoylcarbocyanine iodide (JC-1) dye. In detail, cells (n = 6) were seeded in a 96-well black fluorescence plate (Corning^®^, New York, NY, USA) at a density of 10,000 cells/well and allowed to grow to 80% confluence. After performing the exposures as described above, the wells were washed with PBS and 100.0 μL of a JC1 solution (2.0 μg/mL) was added and incubated for 30 min at 37 °C. At the end of the incubation time, the wells were washed three times with PBS enriched in Ca^2+^ and Mg^2+^ and 100.0 μL of fresh medium was placed in each well for fluorescence reading. To quantify JC1 monomers and J-aggregates fluorescence was read at 485/530 nm (Ex/Em) and 535/390 nm (Ex/Em) respectively using on a Synergy™ H1 multi-mode microplate reader (BioTek, Winooski, VT, USA). Finally, to quantify the ΔΨm we calculated the JC-1 (J-aggregates/monomers) ratio. Similarly to intracellular ROS, representative images of JC-1 were acquired after seeding TM3 cells on Ibidi μ-slide 8-well plates (Ibidi GmbH, Germany) at a density of 3000 cells/well. After an overnight incubation for cell adherence, the cells were exposed to the experimental conditions. Then, the cells were incubated with the JC-1 solution as previously described and observed using a Zeiss LSM 880 Airyscan confocal microscope (Zeiss, Oberkochen, Germany), equipped with a 63×/1.4 NA Plan-Apochromat oil-immersion objective. Image acquisition was controlled by the ZEN software 2.3 (Zeiss), using appropriate laser lines and filter settings for JC1.

### 2.9. Preparation for DNA, RNA and Protein Extraction

Nucleic material was extracted from Leydig cells following the instructions of the NZY DNA/RNA/Protein Isolation Kit (NZYtech, Lisbon, Portugal). DNA and RNA quantification was performed in NanoDrop™ 2000 Spectrophotometers (ThermoFisher Scientific, Carlsbad, CA, USA). Subsequently, to obtain cDNA, RNA was reverse transcribed using the NZY First Stand cDNA Synthesis Kit (NZYtech, Lisbon, Portugal) using 0.25 μg RNA of each sample as starting material. A second cellular pellet was subjected to lysis using protein extraction buffer containing HEPES (10.0 mM), EGTA (0.5 mM), 1% Triton X-100, PMSF (10.0 mM), sodium orthovanadate (10.0 mM) and a protease inhibitor cocktail (Halt Protease & Phosphatase Inhibitor, ThermoFisher Scientific, Carlsbad, CA, USA). After a 10 min incubation, the obtained lysate was centrifuged for 20 min at 14,000× *g* at 4 °C. The instructions given in the BCA Protein Assay Kit (Fisher Scientific, Carlsbad, CA, USA) were followed to quantify the protein concentration in each sample.

### 2.10. Mitochondrial Biogenesis and Steroidogenesis Analysis

Quantitative real-time reverse transcription polymerase chain reaction (RT-qPCR) was performed to analyze the mitochondrial DNA abundance of *NADH dehydrogenase 1* (*MT-ND1*) used as a marker of mtDNA copy number, using *β2-microglobulin* (single copy gene) to normalize the results. In brief, to each sample containing 0.066 μg of cDNA, Power Track SYBR Green Master Mix (Thermo Fisher Scientific, Carlsbad, CA, USA) was added to a solution containing primers, and the final volume was adjusted with RNase-free water up to 20.0 μL/well. RT-qPCR was also used to assess the mRNA expression abundance of enzymes directly involved in androstenedione production (*Star*, *Cyp17a1*; nuclear receptor subfamily 0 group B member 2, *Nr0b2*), as described above. All the experiments were conducted in triplicate and the average was used to calculate the gene abundance using the model proposed by Pfaffl $\left(2^{-\Delta Ct}\right)$ [34]. All primers used for the experiments are shown in Supplementary Table S1.

### 2.11. Detection of Lipid Peroxidation

After extracting the proteins as indicated above, peroxidation detection was performed by slot blot, as indicated by Moreira and collaborators [35]. For each sample 2.5 μg of protein was loaded onto a Hybri-Slot Manifold System (Biometra, Gottingen, Germany) and transferred onto a nitrocellulose (Amersham™ Protran™, GE Healthcare, Munich, Germany) membrane. After transference onto the membrane, it was incubated with 5% non-fat milk blocking solution for 90 min and then incubated with the primary anti-4-hydroxynonenal antibody (1:5000, AB5605) overnight at 4 °C. Finally, the membrane was incubated for 90 min at RT with mouse anti-goat IgG-HRP (1:5000, Sc-2354, Santa Cruz Biotechnology, Inc., Dallas, TX, USA.) to quantify lipid peroxidation groups. Membranes were incubated with WesternBright™ ECL, and image acquisition was performed using the Chemidoc MP Imaging System from Bio-Rad (Hercules, CA, USA). The intensity of each band was quantified using Image Lab 5.1 software from Bio-Rad (Hercules, USA) and the results were normalized to total protein staining (Ponceau S).

### 2.12. Total OXPHOS Complex Protein Levels

We performed Western blot to quantify the expression of mitochondrial transport chain complexes, as reported by Moreira with minor modifications [36]. Specifically, 35 μg of protein sample were dissolved in Laemmli buffer and incubated for 15 min at RT. Subsequently, the samples were resolved using 15% SDS-PAGE at 90 V until complete protein separation. Protein transfer into nitrocellulose membranes was performed using the Trans-Blot^®^ Turbo™ Transfer System (Bio-Rad; 1.3 A, up to 25 V, 10 min). To confirm protein transfer we used Ponceau S staining and then the membrane was incubated for 180 min with a 5% non-fat milk solution to prevent nonspecific antibody binding. Finally, the membranes were incubated overnight at 4 °C using the primary antibody Total OXPHOS Rodent WB Antibody Cocktail (1:500, ab110413, Abcam, Cambridge, UK), and goat anti-mouse IgG (H + L) HRP-conjugated (1:1000, AP308P, Merck, Darmstadt, Germany) was used as secondary antibody. Finally, the protein bands were visualized using WesternBright™ ECL and captured for quantification using the Chemidoc MP Imaging System from Bio-Rad (Hercules, USA). Protein quantification and normalization to total protein staining (Ponceau S) was performed using Image Lab Software 5.1 (Bio-Rad).

### 2.13. Ferric Reducing Antioxidant Power (FRAP) Assay

The colorimetric FRAP assay is one of the most widely used *in vitro* assays to assess the total antioxidant potential. After seeding and exposing the cells in 100 mm Petri dishes as described previously, FRAP was performed as indicated by Patanè and colleagues, with minor modifications [25]. In detail, the FRAP working solution was prepared by combining a solution of acetate buffer (300.0 mM, pH 3.6), FeCl_3_ and a 10.0 mM solution in 40.0 mM HCl of 2,4,6-tripyridyl-s-triazine (TPTZ) in a ratio of 10:1:1 (*v*/*v*/*v*). In a 96-well plate, 180.0 μL of the FRAP working solution and 6.0 μL of the collected media from each sample and PBS were added. After 40 min of incubation in the dark at RT, the absorbance was read at 595 nm using a microplate reader (Multiskan GO, Thermo Fisher Scientific, Vantaa, Finland) to quantify the reduction of the Fe^3+^-TPTZ complex into the blue coloured Fe^2+^-TPTZ complex. A fresh solution of 1.0 mM ascorbic acid was used as a reference standard. The experiments were performed in triplicate for each condition, and the absorbance of the samples was corrected using the blank.

### 2.14. Competitive Elisa for Androstenedione Quantification

To assess the effect of the anthocyanin-enriched fraction on Leydig cell steroidogenesis, we used the *Androstenedione ELISA Kit* (Wuhan Fine Biotech Co., Ltd), a competitive ELISA assay for the steroidogenesis intermediate. In fact, TM3 cells, like other Leydig cell lines (mLTC-1, MA-10, and BLTK1), express low levels of the 17β-HSD3 enzyme, which catalyzes the reduction of androstenedione to testosterone [37]. Thus, the production of androstenedione is used as a marker of steroidogenesis. The media from cells co-exposed to the experimental conditions and hCG were used to measure total androstenedione, following the manufacturer’s protocol. Samples and standards were processed as indicated by the manufacturer, and the resulting concentrations were calculated using CurveExpert software 1.4 (FineTest, USA) and expressed in ng/mL.

### 2.15. Exometabolome Analysis via ^1^H-NMR Spectroscopy

To acquire the ^1^H-NMR spectrum for exometabolome analysis of the medium from each group, we followed the protocol described by Alves and colleagues [38]. In detail, the medium of each sample was centrifuged at 5000× *g* for 5 min and a mixture containing 180.0 μL of media samples and 1.0 mM of sodium fumarate was transferred to an NMR tube. Acquisition of ^1^H-NMR spectra was performed using a Bruker Avance III HD 500 MHz spectrometer equipped with a 5 mm Broadband Inverse (BBI) probe (Bruker Corporation, Billerica, MA, USA). Solvent-suppressed 1D−^1^H-NMR spectra were acquired using a NOESYPR1D pulse sequence, with a sweep width of 7 kHz, relaxation delay of 7 s, pulse angle of 30°, acquisition time of 2.3 s, and 64 scans. The untargeted metabolites identification and characterization were performed by Chenomx profiler software (NMR suite, version 8.3), in accordance with the Metabolomics Standards Initiative guidelines for metabolite identification [39]. Spectra were processed by applying a 0.2 Hz line broadening prior to Fourier transformation, followed by manual phasing and baseline correction. Chemical shifts and metabolite quantification were referenced to sodium fumarate (6.50 ppm), using the Bruker TopSpin software version 4.5.0. Data are expressed as metabolite concentrations (μmol/10^6^ cells). The levels of the identified metabolites are presented in Supplementary Material Table S2. Metabolomic analyses were carried out with the MetaboAnalyst 6.0 program (available on: https://www.metaboanalyst.ca/) (accessed on July 2025), where partial least squares discriminant analysis (PLS-DA), sparse partial least squares discriminant analysis (s-PLSDA), and heatmaps analysis were performed. In addition, quantitative enrichment analysis (QEA) was conducted using the “Enrichment Analysis” module of MetaboAnalyst, with metabolite concentrations as input and mouse pathway libraries based on KEGG. The overview of the top enriched metabolite sets is provided as Supplementary Material Table S2.

### 2.16. Statistical Analysis

Statistical evaluation was conducted using a one-way analysis of variance (ANOVA) under parametric assumptions, followed by Fisher’s Least Significant Difference (LSD) test after confirming data normality. Significance was set at *p* < 0.05. All statistical computations were carried out with GraphPad Prism software, version 8.4.2 (GraphPad Software Inc., San Diego, CA, USA). Multivariate data exploration was performed through principal component analysis (PCA) and partial least squares discriminant analysis (PLS-DA). Differences in metabolite levels among the experimental conditions were further examined using univariate ANOVA coupled with Fisher’s post hoc test, applying a significance threshold of *p* < 0.05. Both multivariate and univariate analyses were executed using MetaboAnalyst version 6.

## 3. Results

### 3.1. Analytical Identification and Quantification of the Anthocyanin-Enriched Fraction via RP-HPLC-DAD-ESI-MS/MS

**Figure 1:**
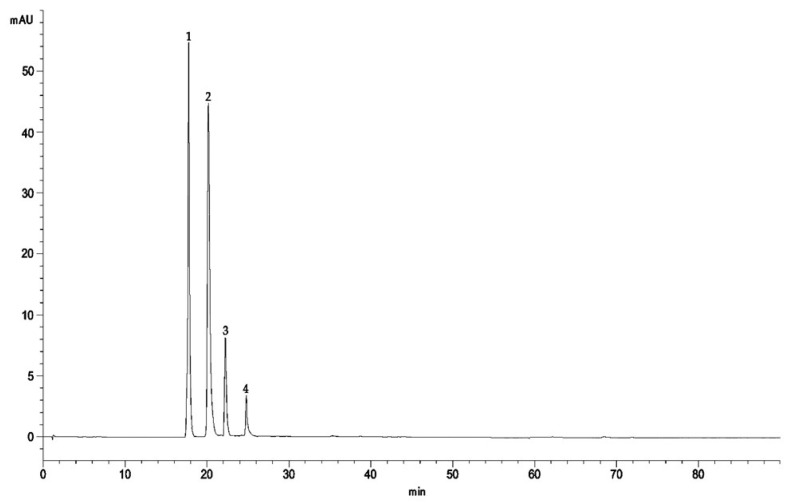
Representative RP-HPLC-DAD-ESI-MS/MS chromatogram of enriched fraction of *Callistemon citrinus* flowers acquired at 520 nm. 1 cyanidin-3,5-O-diglucoside (cyanin), 2 peonidin-3,5-O-diglucoside (peonin), 3 cyanidin-3-O-glucoside, 4 cyanidin-coumaroylglucoside-pyruvic acid.

The comprehensive characterization of the anthocyanin profile obtained from *Callistemon citrinus*-enriched fraction, through reverse-phase high-performance liquid chromatography coupled with diode array and electrospray ionization tandem mass spectrometry (RP-HPLC-DAD-ESI-MS/MS), is depicted in Figure 1.

The chromatographic analysis revealed a polyphenolic composition dominated by anthocyanins, as evidenced in the HPLC-DAD chromatograms acquired at 520 nm. No other peaks were identified at 260, 292, 330, 370 nm, corresponding to the maximum of absorbance of other polyphenols subclasses. Detailed inspection of the UV–Vis spectra together with the MS and MS/MS fragmentation data allowed the identification of four distinct anthocyanin compounds, which were subsequently quantified and expressed as cyanidin-3-O-glucoside (CyG) equivalents (Table 1) according to the data present in the literature [28]. Among them, peonidin and cyanidin derivatives (cyanidin-3,5-O-diglucoside and peonidin-3,5-O-diglucoside) are, by far, the most abundant compounds, followed by cyanidin-3-O-glucoside, and a structurally more complex cyanidin-coumaroylglucoside-pyruvic acid derivative, which represents less than 3% of the total amount of detected anthocyanins.

### 3.2. Cytotoxicity Evaluation of the Anthocyanin-Enriched Fraction on Leydig Cells

**Figure 2:**
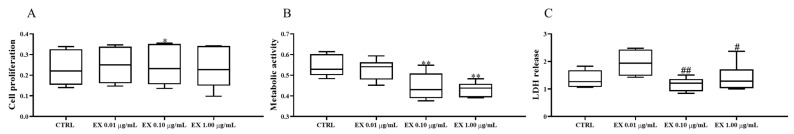
Cytotoxicity profile of Leydig cells subjected to anthocyanins-enriched fraction (0.01, 0.10 and 1.00 µg/mL) through the following assays: (**A**) Cell proliferation; (**B**) Metabolic activity; (**C**) LDH release due to cell membrane permeability damage. Statistically significant differences are indicated above each condition as follows: * *p* < 0.05 vs. CTRL (0 μg/mL of anthocyanins-enriched fraction); ** *p* < 0.01 vs. CTRL; # *p* < 0.05 vs. 0.01 μg/mL of anthocyanins-enriched fraction; ## *p* < 0.01 vs. 0.01 μg/mL of anthocyanins-enriched fraction. Data are presented as box-and-whisker plots (median and minimum-maximum; n = 6). Abbreviations: CTRL: Control; EX: anthocyanins-enriched fraction; SRB: Sulforhodamine; MTT: 3-(4,5-dimethylthiazol-2-yl)-2,5-diphenyltetrazolium bromide; LDH: Lactate dehydrogenase.

We initiated our approach by assessing cell proliferation after exposing Leydig cells with different concentrations of anthocyanin-enriched fraction. We observed an increase in proliferation when Leydig cells were incubated with the anthocyanin-enriched fraction of 0.10 μg/mL (0.24 ± 0.039 Abs) compared to the control (0.23 ± 0.036 Abs) (Figure 2A). No other significant changes in proliferation activity of Leydig cells exposed to the other concentrations of the anthocyanin-enriched fraction were detected. Leydig cell metabolic activity was significantly decreased by the two highest concentrations of the anthocyanin-enriched fraction (0.10 μg/mL: 0.44 ± 0.03 Abs; 1.00 μg/mL: 0.53 ± 0.02 Abs), relative to both the control (0.54 ± 0.02 Abs) and the lowest concentration (0.43 ± 0.02 Abs). Finally, our data show no statistical differences in the release of LDH in Leydig cells after exposure to any of the anthocyanin-enriched fraction compared to control condition. However, exposure of Leydig cells to the lowest concentration tends to increase the release of LDH (1.95 ± 0.191 Abs) (*p* < 0.1) compared to the control (1.35 ± 0.13 Abs). The increase in LDH release by Leydig cells reaches statistical difference when compared with the exposure to the other concentrations of anthocyanin-enriched fraction 0.10 μg/mL (1.17 ± 0.100 Abs) and 1.00 μg/mL (1.41 ± 0.20 Abs).

### 3.3. Exposure to Anthocyanin’s Enriched Fraction Increases Intracellular ROS Production in Leydig Cells

**Figure 3:**
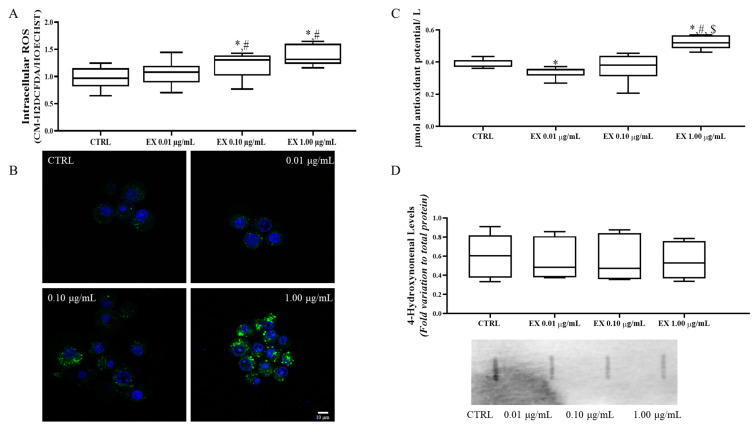
Analysis of redox profile of Leydig cells exposed to anthocyanins-enriched fraction using: (**A**) Intracellular ROS assessed by CM-H_2_DCFDA assay; (**B**) Representative images of CM-H_2_DCFDA assay, with the green and blue fluorescences corresponding to CM-H_2_DCFDA and HOECHST, respectively; (**C**) Antioxidant potential of the collected medium assessed by FRAP assay; (**D**) 4-Hydroxynonenal protein levels assessed by slot blot and representative image of the obtained bands. Data are presented as box-and-whisker plots (median and minimum-maximum; n = 6). Statistically significant differences are indicated above each condition as follows: * *p* < 0.005 vs. CTRL (0 μg/mL anthocyanins-enriched fraction); # *p* < 0.05 vs. 0.01 μg/mL of anthocyanins-enriched fraction; $ *p* < 0.05 vs. 0.10 µg/mL anthocyanin-enriched fraction. Abbreviations: CTRL: Control; EX: anthocyanins-enriched fraction; CM-H_2_DCFDA: 5-(and-6)-chloromethyl-2′,7′-dichlorodihydrofluorescein diacetate; FRAP: Ferric reducing antioxidant power.

Anthocyanins are known for their antioxidant power, but several studies also highlight their pro-oxidant potential at specific concentrations and in different tissues and cells [40,41]. Therefore, we measured intracellular ROS production in Leydig cells using CM-H2DCFDA/HOECHST fluorescence. As shown in Figure 3A, exposure to the anthocyanin-enriched fraction increases intracellular Leydig cells ROS production in a dose-dependent way. In detail, exposure of Leydig cells to 0.10 μg/mL of anthocyanin-enriched fraction (1.21 ± 0.10 Abs) increased ROS production compared to the control (0.97 ± 0.09 Abs), and linearly, the exposure of Leydig cells to the highest concentration of anthocyanin-enriched fraction 1.00 μg/mL (1.39 ± 0.09 Abs) further increased ROS levels compared to the control. Furthermore, exposure of Leydig cells to both concentrations also show a significant increase compared to the lowest concentration of 0.01 μg/mL (1.06 ± 0.10 Abs). These results were further confirmed by fluorescence imaging (Figure 3B). In Figure 3C, we assessed the total antioxidant potential of the collected media after the exposure to the experimental conditions. The media of Leydig cells exposed to the lowest concentration of anthocyanin-enriched fraction 0.01 μg/mL (0.34 ± 0.02 Abs) showed a significant decrease in antioxidant potential compared to the control (0.39 ± 0.01 Abs), while the media of Leydig cells exposed to the highest concentration 1.00 μg/mL (0.52 ± 0.02) showed a significant higher antioxidant potential compared to the control group. One of the most important ROS-associated damages is lipid peroxidation. Thus, we evaluated lipid peroxidation by measuring the 4-hydroxynonenal. As shown in Figure 3D, no significant changes were observed in Leydig cells lipid peroxidation after exposure to any of the anthocyanin-enriched fraction compared to the control.

### 3.4. Exposure to the Lowest Concentration of Anthocyanin-Enriched Fraction Impacts Mitochondrial Quality

**Figure 4:**
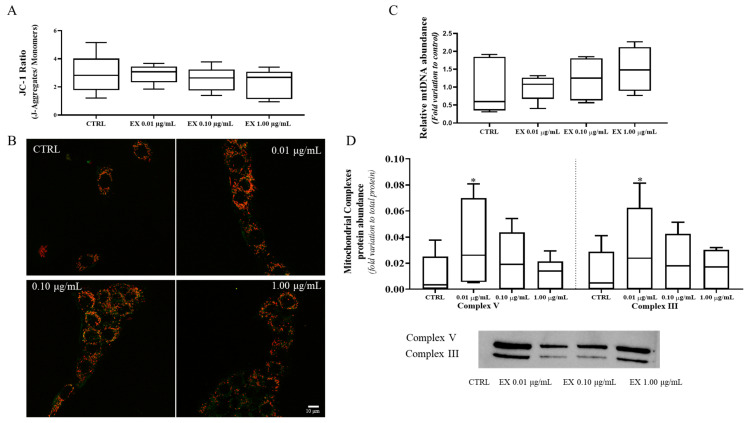
Evaluation of mitochondrial state in Leydig cells exposed to anthocyanins-enriched fraction (0.01, 0.10 and 1.00 µg/mL) using: (**A**) Mitochondrial membrane potential represented by the ratio aggregates/monomers obtained in the JC-1 assay; (**B**) Representative images of mitochondrial membrane potential changes following exposure to anthocyanin-enriched fraction with the red and green fluorescences corresponding to JC-1 J-aggregates and monomers, respectively; (**C**) Relative abundance of mtDNA copy number; (**D**) Protein levels of mitochondrial complex V and III of oxidative phosphorylation. Data are presented as box-and-whisker plots (median and minimum-maximum; n = 6). Statistically significant differences are indicated above each conditions as follows: * *p* < 0.05 vs. CTRL (0 µg/mL anthocyanin-enriched fraction). Abbreviations: CTRL: Control; EX: anthocyanins-enriched fraction; JC1: 1-5,5,6,6′-tetrachloro-1,1′,3,3,3′ tetraethylbenzyme-dazoylcarbocyanine iodide.

Although not statistically significant (*p* > 0.05), a dose-dependent decrease in ΔΨm was observed with increasing concentrations of the anthocyanin-enriched fraction (Figure 4A). In particular, the Leydig cells exposure to the highest concentration of 1.00 μg/mL (2.31 ± 0.41) yielded a nearly significant difference versus control (2.88 ± 0.64; *p* = 0.07), suggesting a potential dose–response relationship. The analysis of ΔΨm was also observed by the acquisition of fluorescence imaging (Figure 4B). A similar dose-dependent trend was observed for total mitochondrial copy number which increased with higher concentrations of the anthocyanin-enriched fraction, although this effect did not reach statistical significance (Figure 4C). To better understand how anthocyanin-enriched fraction affects mitochondria physiology, we measured the levels of oxidative phosphorylation complexes. We detected that Leydig cells exposed to the lowest concentration of anthocyanin-enriched fraction (0.01 μg/mL) present an increase in the levels of complex V (0.04 ± 0.01—fold variation to total protein) and complex III (0.03 ± 0.01—fold variation to total protein) compared to their respective controls (0.01 ± 0.007 and 0.013 ± 0.007—fold variation to total protein) (Figure 4D).

### 3.5. Higher Concentrations of Anthocyanin-Enriched Fraction Inhibit Steroidogenesis and Androstenedione Production in Leydig Cells

**Figure 5:**
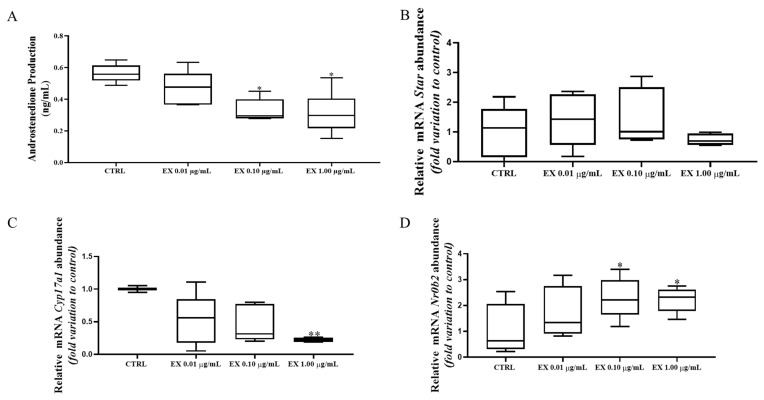
Impact of anthocyanin-enriched fraction on steroidogenesis. (**A**) Evaluation of androstenedione production; Evaluation of mRNA expression of steroidogenic acute regulatory (Star) (**B**), Cytochrome P450 17A1 (Cyp17a1) (**C**) and small heterodimer partner (Nr0b2) (**D**), respectively normalized to β2-microglobulin and expressed as fold change relative to control. Data are presented as box-and-whisker plots (median and minimum-maximum; n = 6). Statistically significant differences are expressed as follow: * *p* < 0.05 vs. CTRL (0 µg/mL anthocyanin-enriched fraction; ** *p* < 0.01 vs. CTRL. Abbreviations: CTRL: Control; EX: anthocyanins-enriched fraction.

As mentioned, using our *in vitro* model we can measure androstenedione release, the precursor of testosterone, in culture medium. As shown in Figure 5A, Leydig cells exposure to 0.10 μg/mL (0.33 ± 0.03 ng/mL) and 1.00 μg/mL (0.31 ± 0.05 ng/mL) of the anthocyanin-enriched fraction resulted in a significant, nearly dose-dependent decrease in androstenedione production compared with the control group (0.56 ± 0.03 ng/mL). To further investigate the effect of the anthocyanin-enriched fraction in steroidogenesis, we evaluated the expression of key steroidogenic genes. Star mRNA expression was not affected by the exposure to any of the anthocyanin-enriched fraction concentrations tested (Figure 5B). In contrast, the exposure of Leydig cells to the highest concentration of anthocyanin-enriched fraction (1.00 μg/mL: 0.22 ± 0.018) significantly reduced Cyp17a1 mRNA expression compared with the control (1.00 ± 0.030) (Figure 5C). To better understand the decrease in androstenedione production, we also analyzed Nr0b2, a transcription factor known to act as a repressor of steroidogenesis. Its high expression has been inversely correlated with the expression of Star, Cyp11a1, 3βhsd and Cyp17a1 [42]. In our study, Nr0b2 mRNA expression was significantly increased after Leydig cells exposure to 0.10 μg/mL (2.29 ± 0.35) and 1.00 μg/mL of anthocyanin-enriched fraction (2.22 ± 0.22) compared with the control (1.00 ± 0.52) (Figure 5D).

### 3.6. Exometabolome Analysis

**Figure 6:**
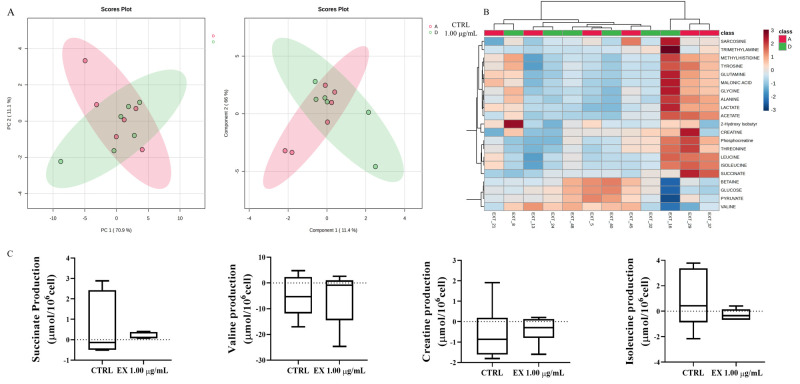
Multivariate and univariate analysis of exometabolomic alterations in Leydig cells exposed to the highest concentration of anthocyanin-enriched fraction. (**A**) PCA and PLS-DA score plots showing partial overlap but clear separation between control and exposed groups with 1.00 μg/mL of anthocyanins-enriched fraction, indicating distinct metabolic profiles. (**B**) Hierarchical clustering heatmap displaying differential metabolite abundance, with control samples clustering separately from exposed cells, consistent with an anthocyanin-induced metabolic shift. (**C**) Box and whisker plots of representative metabolites showing increased extracellular succinate, valine, and creatinine. Data are expressed as normalized peak areas (unit variance scaling); n = 6 biological replicates per groups. Abbreviations: CTRL: Control; EX: anthocyanins-enriched fraction.

To evaluate the global alterations in the exometabolomic profile, a multivariate analysis was performed. PCA, an unsupervised method, was initially applied to visualize the natural separation between the groups. The PCA scores plot shows a tendency for clustering but with considerable overlap between the control group (A) and the group exposed to the highest concentration of anthocyanin-enriched fraction (D), indicating no clear separation based on the first two principal components (PC1: 70.9%, PC2: 11.1%), as can be seen in Figure 6A. To maximize the distinction between the groups, a supervised PLS-DA was subsequently performed. The PLS-DA scores plot demonstrates a more distinct and clear separation between the metabolic profiles of groups A and D. The supervised PLS-DA model successfully captured consistent and significant metabolic differences induced by the highest concentration of the anthocyanin-enriched fraction in Leydig cells, which were not resolved by the unsupervised PCA (Figure 6A). To further visualize the differences in the exometabolomic profiles, a hierarchical clustering analysis was performed, and the results are presented as a heatmap in Figure 6B. The analysis reveals a distinct separation of the samples based on their metabolic signatures. The samples from the control group (A) cluster together on the right side of the heatmap are clearly distinguished from most of the samples from Leydig cells with the highest concentration of the anthocyanin-enriched fraction (D), which cluster on the left. This indicates a significant and consistent metabolic shift induced by the extract. Specific patterns of metabolite abundance are evident. A large group of metabolites, including numerous amino acids (e.g., sarcosine, glutamine, glycine, alanine, leucine, isoleucine) and organic acids (e.g., lactate, succinate), show a markedly higher relative abundance in the extracellular medium of the control cells (Group A), as indicated by the red and orange colours. Overall, the heatmap confirms a clear distinct metabolic phenotype where control cells display high consumption of glucose and pyruvate, coupled with high secretion or accumulation of various amino acids and lactate, a profile that is significantly attenuated in the cells exposed to the highest concentration of the anthocyanin-enriched fraction (Group D). The box plots illustrate the relative extracellular concentrations of metabolites that exhibited the variations between control Leydig cells (Group A) and those exposed to the highest concentration of the anthocyanin-enriched fraction (Group D). The data show a distinct metabolic shift after Leydig cells exposure to the anthocyanin-enriched fraction (Figure 6C). The extracellular concentrations of the branched-chain amino acid valine, creatinine, along with the tricarboxylic acid cycle (TCA) cycle intermediate succinate, were higher in the medium of the exposed Leydig cells (Group D) compared to the control group (Group A). Conversely, the extracellular concentration of isoleucine was lower in the exposed group. In conclusion, these findings point to a possible selective metabolic reprogramming of Leydig cells induced by the highest anthocyanin-enriched fraction, characterized by an enhanced consumption of specific amino acids and energy substrates, coupled with alteration in isoleucine. In addition to multivariate and univariate analyses, a QEA was performed to investigate whether the observed metabolite changes aggregated into significantly enriched metabolic pathways. Altough several pathways related to amino acid metabolism and energy production were among the top-ranked sets, none of them reached statistical significance (*p* value > 0.05), indicating the absence of robust, pathway-level enrichment under the present conditions. For this, the complete QEA representation is provided in Supplementary Material Table S2.

## 4. Discussion

Anthocyanins are water-soluble polyphenolic compounds abundant in Mediterranean diets, widely studied for their antioxidant and anti-inflammatory properties. Although the estimated dietary intake of anthocyanins can reach approximately ~200 mg/day, their bioavailability remains low (1–10%), depending on the type of aglycone, degree and type of glycosylation [31]. Consequently, biological effects depend not only on their dietary intake but also on their effective cellular concentration and metabolic transformation. Interestingly, anthocyanins can exert a concentration-dependent biphasic behaviour: at low doses they act as antioxidants, while at higher doses they shift toward pro-oxidant effects depending on the redox environment and cellular context [40,43]. This paradox challenges the traditional view of anthocyanins as universally beneficial and explains their ability to either increase intracellular ROS or activate protective pathways—effects central to understanding their impact on endocrine function. Nowadays, OS is widely recognized as contributing approximately 30–80% to male infertility [44]. This strong correlation underscores the importance of dietary factors as potential modulators of testicular redox balance [13]. However, despite their widespread use as reproductive health supplements, the effects of anthocyanins under non-pathological conditions remain unexplored in steroidogenic cells. Previous investigations focused exclusively on pathological models, using oxidative stressors (e.g., AAPH, heavy metals, pollutants) to induce conditions under which anthocyanins restored androgen production—highlighting their therapeutic potential in diseased states [45,46,47]. Critically, these studies did not address the molecular mechanisms underlying anthocyanin modulation of steroidogenesis in normal, unstressed Leydig cells, nor did they clarify whether dietary anthocyanin intake at physiologically relevant concentrations could modulate androgen production independently of external stressors. This gap is clinically important: evidence-based dietary recommendations for male reproductive health require knowledge of anthocyanin effects under normal conditions. To clarify the mechanistic role of anthocyanins in androgen production, this study examined an anthocyanin-enriched fraction from *Callistemon citrinus* using TM3 Leydig cells as an *in vitro* steroidogenesis model. Initial assessment examined the impact of anthocyanin exposure (24 h) on cell proliferation, metabolic activity, and LDH release across relevant concentrations (0, 0.01, 0.10, 1.00 μg/mL). The lowest concentration (0.01 μg/mL) of anthocyanin-enriched fraction did not affect any of the tested parameters. In contrast, the higher concentrations (0.10 and 1.00 μg/mL) reduced metabolic activity by the MTT assay, while 0.10 μg/mL also promoted a significant increase in cell proliferation compared to the unexposed control. Redox profile analysis revealed the biphasic antioxidant/pro-oxidant dualism of anthocyanins [40]. FRAP assay on the culture media demonstrated that the lowest concentration of anthocyanin-enriched fraction significantly reduced its antioxidant potential compared to the control, likely due to metal complexation or auto-oxidation processes [48]. Conversely, the highest concentration increased the reducing capacity, probably for the higher availability of phenolic hydroxyl groups. However, the results obtained using CM-H_2_DCFDA demonstrated that both 0.10 and 1.00 μg/mL increased intracellular ROS production in a near dose-dependent way. Interestingly, despite this ROS production, slot blot analysis detected no lipid peroxidation in cell membranes, suggesting a controlled oxidative response. This biphasic profile reflects anthocyanin behaviour: at lower concentrations they act as antioxidant, while at higher concentrations they shift to pro-oxidant effects, particularly *via* Fenton reaction generating redox-active complexes, quinones, semiquinones and H_2_O_2_. Structure–activity relationship studies further indicate that anthocyanins such as cyanidin, delphinidin and pelargonidin display stronger pro-oxidant than antioxidant activity thanks to the free hydroxyls group on the B aromatic ring [40,49]. Beyond their biphasic effects, anthocyanins, and flavonoids in general, can be classified as hormetic compounds, capable of eliciting adaptive stress responses. In this framework, mild OS may activate adaptive cellular responses that mitigate ROS-mediated damage, preserving redox homeostasis and membrane integrity. Supporting this findings, slot blot data indicates that without excessive ROS accumulation, Leydig cells maintain defensive mechanisms preventing lipid peroxidation and explaining why no LDH release was detected despite elevated intracellular ROS [50]. The observed adaptive response can be explained by the Keap1-Nrf2 pathway. Under physiological conditions, Keap1 (Kelch-like ECH-associated protein 1) keeps Nrf2 inactive in the cytoplasm [51]. During OS-conditions, Keap1 cysteine oxidation release Nrf2, which translocates to the nucleus and binds Antioxidant Response Elements (ARE). This enhances transcription of antioxidant enzymes (SOD, CAT, Heme-oxygenase 1 (HO-1), glutathione peroxidase (GPx), and NAD(P)H dehydrogenase (NQO1)), preventing lipid peroxidation and cell lysis. Furthermore, it has been demonstrated in Caco-2 cells that cyanidin -3-O-glucoside, a component of the anthocyanin-enriched fraction, can promote Nrf2 nuclear translocation even under physiological conditions and at low concentrations (20/40 μM), thus boosting antioxidant defences independently of external stressors [52]. We further suggest that this pro-oxidant activity represents the primary molecular event initiating the downstream physiological response observed in this study. In fact, given mitochondrial health’s central role in steroidogenesis, Δψm was analyzed after 24 h exposure as an initial indicator of mitochondrial function. JC-1 results did not reveal statistically significant changes; however, the trend was consistent with intracellular ROS data. Specifically, Δψm tended to be higher with the lowest concentration (0.01 μg/mL) of anthocyanin-enriched fraction, while at higher concentrations it decreased compared with the control, in parallel with the ROS increase. These findings suggest that the adaptive antioxidant effect previously observed may also occur at the mitochondrial level [53]. Supporting this interpretation, mitochondrial DNA (mtDNA) copy number increased in association with the trend toward mitochondrial depolarization [53]. Here, the mitochondria emerge as the central hub for this hermetic response. At the lowest concentration (0.01 μg/mL), Leydig cells exhibited a marked upregulation of oxidative phosphorylation complexes III (ubiquinone-cytochrome c reductase) and V (ATP synthase) in the absence of any detectable ROS increase. This is a hallmark of mitohormesis, an adaptive mechanism in which a mild redox stimulus enhances mitochondrial resilience and bioenergetic efficiency. Complex III, together with complex I, represents a major site of superoxide (O_2_^−^•) generation during electron transfer from ubiquinol to cytochrome c and proton translocation into the intermembrane space. Its stimulation at the lowest concentration (0.01 μg/mL) may help to maintain ΔΨm while limiting oxidative damage caused by ROS produced at this site. In contrast, complex V is not directly involved in ROS production but is essential for ATP synthesis. The observed stimulation of complex V by low anthocyanin-enriched fraction suggests a pro-energetic effect, reflected in higher mitochondrial polarization and metabolic efficiency. Conversely, higher concentrations (0.10 and 1.00 μg/mL) failed to stimulate these complexes and instead elicited markers of mitochondrial dysfunction, including a dose-dependent trend to decline Δψm. Mitochondrial depolarization and reduced metabolic activity indicate that the hormetic response has exceeded the adaptive threshold, allowing oxidative phosphorylation to become the primary ROS source via electron leakage. This reveals a biphasic anthocyanin effect: low doses enhance mitochondrial function, while higher doses shift mitochondria from protectors to ROS generators. Such biphasic behaviour aligns with previous evidence showing that several natural flavonoids, including luteolin and related polyphenols, display hermetic responses in which low doses activate protective pathways while higher doses trigger oxidative or metabolic stress [54,55,56]. These results are also in line with previous report showing that elevated ROS levels can induce mitochondrial membrane depolarization by oxidizing complex V and III components or increasing membrane permeability [57]. At the same time, our data point to a compensatory mechanism of mitochondrial hormesis, in which transcription factors such as TFAM, NRF1/2 and PGC-1α may stimulate mitochondrial biogenesis and expansion of the mitochondrial pool to preserve redox homeostasis [58,59]. Clearly, these compensatory mechanisms appear to occur only under sub-toxic ROS conditions. For instance, in MA-10 cells exposed to 250.0 μM H_2_O_2_, a total loss of ΔΨm and a rapid decrease in ATP production (from 34.9 ± 4.0 to 28.7 ± 6.3 pmol ATP/μg protein within 60 min) have been reported, indicating that mitochondrial adaptation is not always possible [57]. Having established the biphasic redox effects of the anthocyanin fraction, subsequent analyses examined how these changes translate into alterations in steroidogenesis, specifically through the quantification of androstenedione, the final precursor of testosterone. This approach was based on the fact that TM3 cells express low concentrations of 17β-HSD3, limiting testosterone production [37]. ELISA quantification demonstrated that anthocyanins at concentrations of 0.10 and 1.00 μg/mL significantly reduced androstenedione production compared to control stimulated only with hCG (3.0 ng/mL) to activate the AC/cAMP axis. This result appears to contrast with previous reports showing that anthocyanins can stimulate or restore steroidogenesis in Leydig cell models [45,46,47]. However, those studies exclusively examined anthocyanin effects under pathological stress conditions (AAPH-induced oxidative stress, acrylamide, cadmium exposure), whereas this work represents the first assessment under basal redox and unstressed conditions. To clarify the basis of the observed decrease, mRNA levels of *Star, Cyp17a1* and *Nr0b2* were analyzed. Changes in steroidogenesis are typically attributed to StAR expression, as this protein represents a rate-limiting step in testosterone biosynthesis by regulating cholesterol transport into mitochondria [60]. Although results did not reach statistical significance, only the highest concentration of anthocyanin-enriched fraction appeared to reduce *Star* expression, whereas lower concentration showed a positive trend. This observation suggests that reduced androstenedione production cannot be solely explained by alterations in StAR expression or ΔΨm, since JC-1 analysis revealed no significant changes in membrane potential. Because StAR is strongly regulated by the AC/cAMP pathway, anthocyanins may indirectly modulate AC activity, leading to increased intracellular cAMP levels, as demonstrated for cyanidin-3-O-glucoside in HEK293 cells [61]. Additionally, low anthocyanin concentrations (25μM) are able to inhibit phosphodiesterase (PDE) activity in COS-7 cells maintaining a more persistent pro-steroidogenic signal in this *in vitro* model [62]. We further studied a molecular pathway that links the pro-oxidant effect of the anthocyanin-enriched fraction to the suppression of androstenedione synthesis. The primary steroidogenic lesion was a significant, dose-dependent downregulation of *Cyp17a1*, the key enzyme in androgen synthesis, while the rate-limiting cholesterol transporter *Star* remained largely unaffected. The most significant mechanistic findings was the concurrent upregulation of the orphan nuclear receptor *Nr0b2* (Small Heterodimer Partner, SHP). This repressor modulates transcription factors such as Steroidogenic Factor-1 (NR5A1) and Liver Receptor Homolog-1 (LRH-1/NR5A2), which normally stimulate enzymes including Star, Cyp17a1 and Cyp11a1 [8]. This provides a coherent chain of events: higher doses of anthocyanin-enriched fraction induce mitochondrial dysfunction and increased intracellular ROS. This ROS signal, acting as a redox-dependent second messenger, may trigger a marked increase in *Nr0b2* transcription. The resulting SHP accumulation then represses its downstream targets, particularly *Cyp17a1*, leading to suppressed enzymatic activity and reduced androstenedione synthesis. Although anthocyanins can enhance cAMP production and could theoretically sustain *Star* expression, our data suggest that this potential pro-steroidogenic effect is overridden by two potential inhibitory mechanisms. First, as previously demonstrated, elevated ROS levels may repress Nur77, a major transcription factor that regulates the expression of steroidogenic enzyme genes, through activation of the c-Jun N-terminal kinase (JNK) pathway, which stimulates *c-Jun* and inhibits *Nur77*-driven transcription [63]. Second, we hypothesize that elevated ROS-induced upregulation of Nr0b2/SHP reinforces transcriptional repression by directly antagonizing SF-1 and LRH-1, both of which are essential steroidogenic transcription factors. Together, these events explain the paradoxical reduction in androstenedione despite potential cAMP-mediated stimulation of StAR. The exometabolome analysis provides critical insight into the cellular metabolic response, supporting a model of a specific, targeted biological effect rather than a global metabolic collapse. PCA and PLS-DA confirmed a significant and consistent metabolic shift at the highest anthocyanin concentration. The heatmap and boxplot data revealed that Leydig cells exposed to the highest concentration of the anthocyanin-enriched fraction exhibited a significantly altered nutrient exchange patterns compared to controls. Specifically, we observed a marked increase in the extracellular concentrations of the branched-chain amino acid valine, the TCA cycle intermediate succinate, and creatine. This indicates a decreased cellular uptake or an increased secretion of these metabolites in response to the anthocyanin-induced stress. This metabolic signature, occurring alongside a general decrease observed in metabolic rate, suggests a metabolic reprogramming. The accumulation of succinate is particularly noteworthy. Succinate accumulation typically signals mitochondrial dysfunction or reversal of the TCA cycle under stress conditions [64]. In this model, extracellular succinate likely serves as a ‘distress signal’, exported by stressed Leydig cells potentially via succinate-specific transporters. Alterations within the TCA cycle lead to succinate accumulation, which diffuse to the cytosol and are released into the extracellular space, through organic anion/dicarboxylate transporters (OATs) to act as a metabolic stress signal [64,65]. In contrast, extracellular isoleucine (a branched-chain amino acid) was significantly lower in Leydig cells exposed to the highest concentration of the anthocyanin-enriched fraction highlighting selective uptake. This differential handling of structurally similar amino acids (valine vs. isoleucine) underscores the specificity of the metabolic rewiring [66]. The absence of major changes in global metabolites like glucose and lactate strongly argues against non-specific toxicity and reinforces that the *Callistemon citrinus* anthocyanin-enriched fraction acts through selective targeting of mitochondrial redox signalling and steroidogenic gene networks [67]. These findings fundamentally challenge the paradigm of phytochemicals as universal antioxidants. Anthocyanins function as potent modulators of intracellular signalling in Leydig cells, capable of eliciting complex, dose-dependent hormetic responses rather than unidirectional antioxidant effects. Although this study employed an *in vitro* system using a complex anthocyanin-enriched fraction, the molecular mechanism proposed herein provides a robust conceptual framework for future research. Critical next steps should include siRNA-mediated knockdown of *Nr0b2* to confirm its causal role in steroidogenic suppression, analysis of upstream ROS-sensitive kinases, and co-treatment with antioxidants such as N-acetylcysteine (NAC) to verify that ROS act as the essential mediators of this pathway. In parallel, direct assessment of CYP17A1 at the protein and enzymatic activity levels will be necessary to validate the contribution of *Cyp17a1* repression beyond the transcriptional level. Additionally, dose–response validation in human Leydig cells and exploration of Nr0b2-indipendent mechanism would strengthen translational relevance. Given that anthocyanins are extensively metabolized, future work should also address the effects of major phase II and degradation products, including protocatechuic acid (PCA), to determine whether these metabolites reproduce or diverge from the steroidogenic and redox responses observed with the parent anthocyanin-enriched fraction.

## 5. Conclusions

This study reveals a biphasic activity of an anthocyanin-enriched fraction derived from *Callistemon citrinus* in Leydig cells. At low concentrations, the fraction triggers adaptive mitohormetic signalling, whereas at higher concentrations it is associated with increased ROS levels and a marked suppression of androgen synthesis, suggesting a redox-dependent mechanism in the observed effects. A possible novel molecular pathway emerges: anthocyanin-induced intracellular ROS activates the transcriptional repressor Nr0b2 (Small Heterodimer Partner), leading to Cyp17a1 downregulation and reduced androstenedione synthesis. Rather than providing definitive proof, our data support a model in which anthocyanin-enriched fraction may negatively modulate steroidogenesis through a redox-sensitive ROS-Nr0b2-Cyp17a1 regulatory axis under basal *in vitro* conditions. If this proposed mechanism is confirmed by future *in vivo* studies or by targeted functional approaches (such as antioxidant co-treatments or direct manipulation of Nr0b2 signalling), it could contribute to reshaping the prevailing perception of dietary antioxidant in the context of male reproductive health, in line with the ‘’antioxidant paradox’’ concept. Moreover, the conservation of Nr0b2/SHP signalling across mammalian species suggests potential translational relevance for human male reproductive health [68]. From a nutritional perspective, these findings carry significant implications. The concentrations tested in this study (0.01–1.00 μg/mL) were selected to fall within a low μg/mL range that is numerically compatible with systemic exposure after high anthocyanin intake or supplementation. Assuming daily intakes up to ~200 mg and an overall bioavailability in the 1–10% range, estimated circulating levels of anthocyanins and their metabolites can reach approximately 0.4–4 μg/mL. In this context, the concentrations used here can be viewed as defining an *in vitro* window of exposure that may overlap with the upper range of systemic levels. While low dietary anthocyanin intake may induce beneficial hormetic responses, excessive intake *via* concentrated supplements or fortified foods may inadvertently suppress androgen production in susceptible individuals. These results underscore the necessity of adopting dose-dependent, context-specific approaches to polyphenol supplementation in reproductive health. This work emphasizes the importance of understanding endocrine-specific mechanisms in dietary interventions, moving beyond simplistic perceptions of phytochemicals as universally beneficial antioxidants.

## Figures and Tables

**Table 1:** Profile of the anthocyanin-enriched fraction obtained from *Callistemon citrinus* by RP-HPLC-DAD-ESI-MS/MS analysis. Results represent the mean ± SD (n = 3) of three independent experiments and are expressed as mg cyanidin-3-O-glucoside equivalents (CyG/100 g of dry extract (DE)).

Peak	Compound	R_t_ (min)	λmax (nm)	MS (*m*/*z*) [M + H]^+^	MS/MS (*m*/*z*) [M + H]^+^	mg CyG/100 g DE
1	Cyanidin 3,5-O-diglucoside	17.92	512	611	449; 287	293.27 ± 2.51
2	Peonidin-3,5-O-diglucoside	20.05	514	625	463; 301	184.31 ± 2.41
3	Cyanidin-3-O-glucoside	22.10	512	449	287	36.84 ± 0.33
4	Cyanidin-coumaroylglucoside-pyruvic acid	24.88	506	661; 595	482	11.57 ± 0.71

## Data Availability

The original contributions presented in this study are included in the article/Supplementary Material. Further inquiries can be directed to the corresponding author.

## Supplementary Materials

The following supporting information can be downloaded at https://www.mdpi.com/article/10.3390/antiox15050530/s1, Table S1. Description of primers for qRT-PCR used in this research. Table S2. Assignment of resonances in the ^1^H-NMR spectra of Leydig cells media and quantitative enrichment analysis (QEA). Figures S1 and S2. Supplementary materials for western/slot blots. Figure S3. Quantitative enrichment analysis (QEA) of exometabolomic data from control Leydig cells (Group A) and cells exposed to the highest concentration of the anthocyanin-enriched fraction (Group D). The dot plot summarizes the pathway-level results obtained using the QEA module implemented in MetaboAnalyst, based on the quantified extracellular metabolites. Each dot represents a metabolic pathway.
