# Supplementary material for: Pro-Oxidant Anthocyanins-Enriched Fraction Inhibits Androgen Synthesis by Transcriptional Repression of Cyp17a1 Through Nr0b2
Source: Antioxidants (Basel). 2026 Apr 23;15(5):530. doi: 10.3390/antiox15050530 (PMC13203438; doi:10.3390/antiox15050530)
Supplement: Supplementary file 1 [file antioxidants-15-00530-s001.zip › Supplementary Materials 3 (blots) 13_04_26.pdf]

Pro-oxidant anthocyanins enriched fraction inhibits androgen synthesis by transcriptional repression of *Cyp17a1* through *Nr0b2*

Giuseppe T. Patanè [a,b,c], Ruben J. Moreira [c,d] , Ana D. Martins [d], Pedro F. Oliveira [d], Stefano Putaggio [a], Davide Barreca [a], Marco G. Alves [*c]

[a] Department of Chemical, Biological, Pharmaceutical and Environmental Sciences, University of Messina, 98166 Messina, Italy

[b] Prof. Antonio Imbesi" Foundation, University of Messina, Messina, 98100, Italy

[c] Institute of Biomedicine, Department of Medical Sciences (iBiMED), University of Aveiro, 3810-193 Aveiro, Portugal

[d] LAQV-REQUIMTE, Department of Chemistry, University of Aveiro, 3810-193 Aveiro, Portugal

*Corresponding Author(s)-Email: Marco G. Alves email: marcoalves@ua.pt telephone: +351967245248

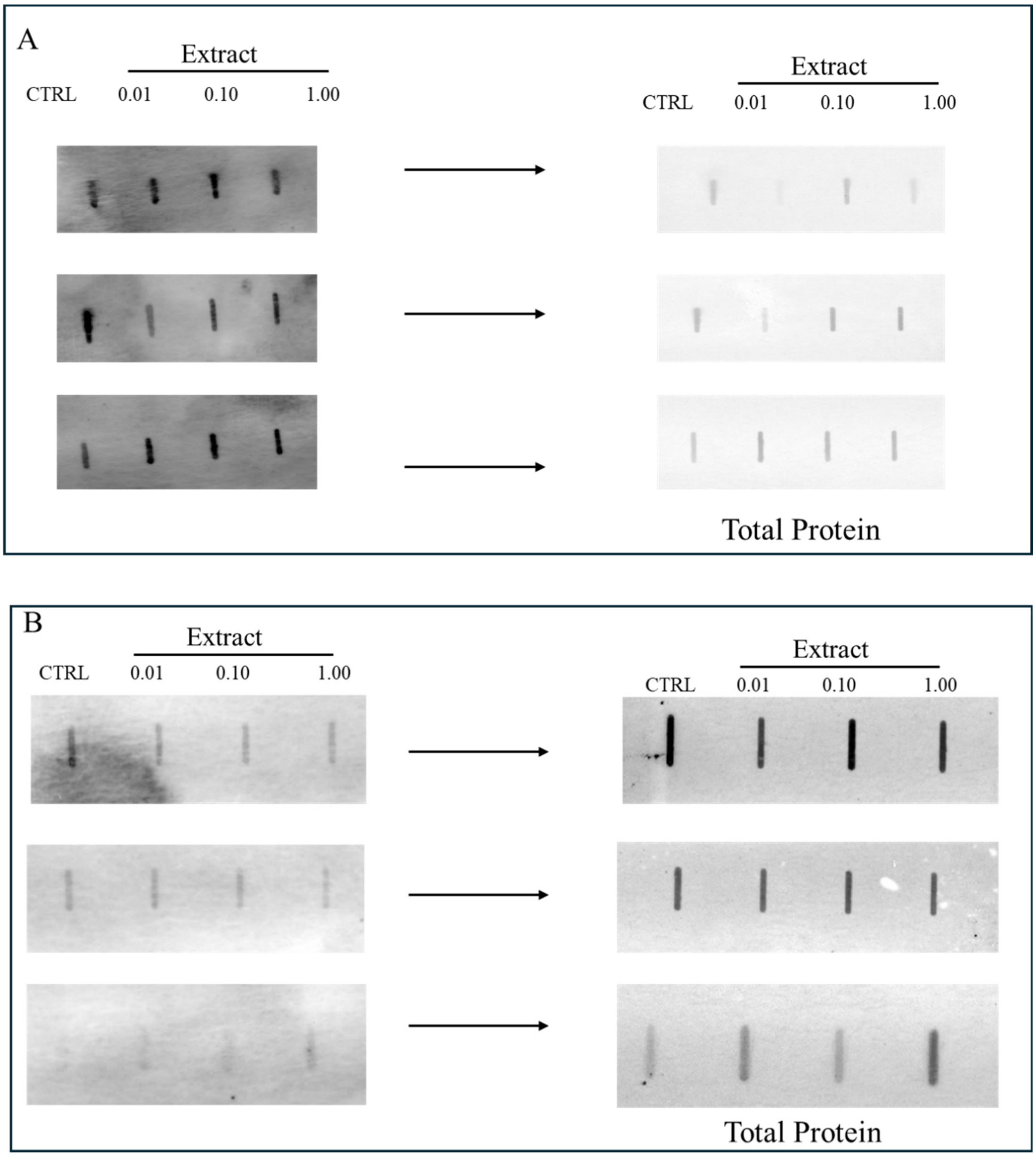

Figure S1. Uncropped image obtained Analysis of lipid peroxidation using as primary antibody anti-4-hydroxynonenal antibody (1:5000, AB5605), in TM3 cells after the exposure to the extract. Panel A correspond to n=1, n=2 and n=3 and respective total protein level. Panel B correspond to n=4, n=5 and n=6 and respective total protein level.

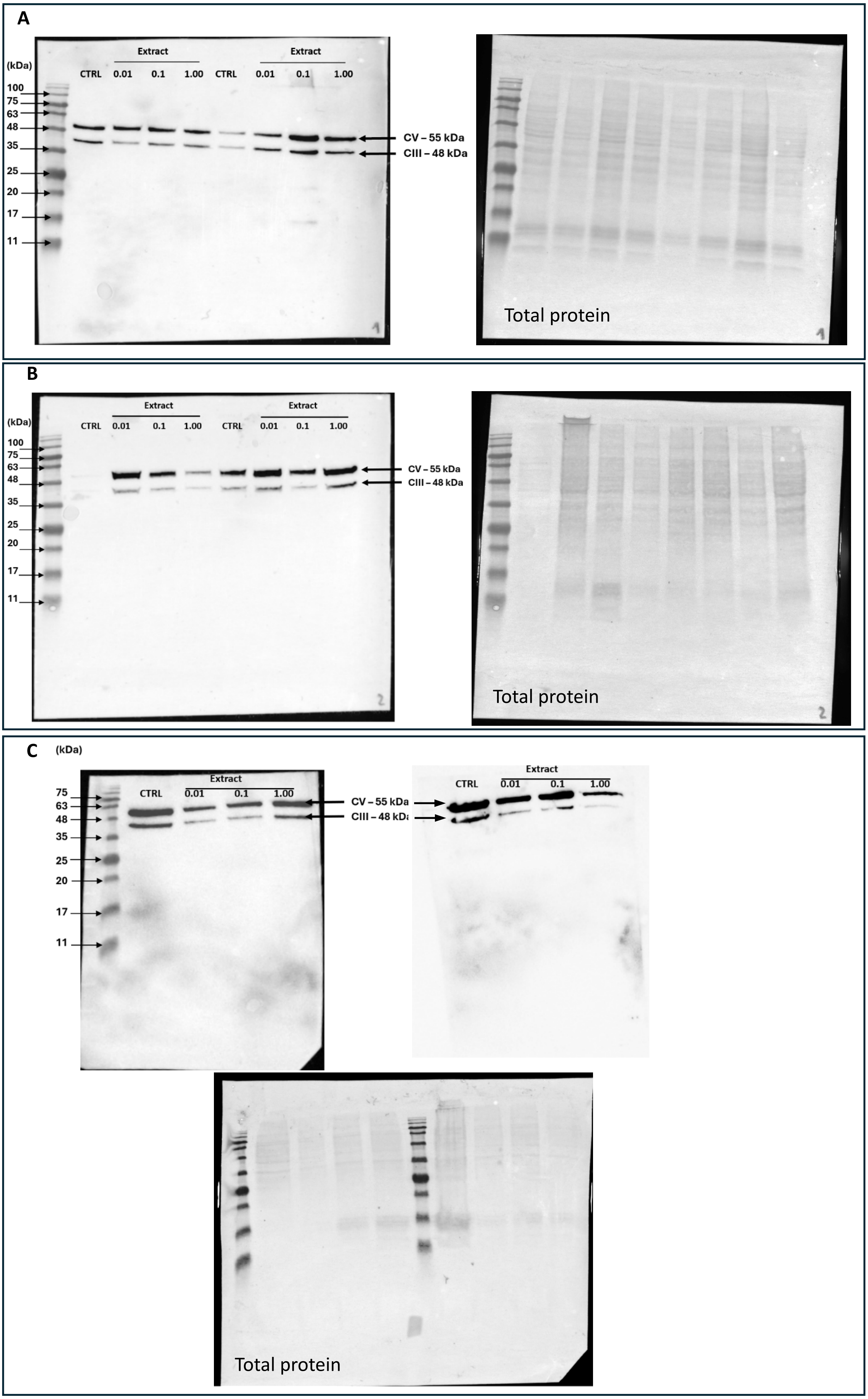
A
(kDa)
Extract
Extract
CTRL
0.01
0.1
1.00
CTRL
0.01
0.1
1.00
100
75
63
48
35
25
20
17
11
CV – 55 kDa
CIII – 48 kDa
Total protein
B
(kDa)
Extract
Extract
CTRL
0.01
0.1
1.00
CTRL
0.01
0.1
1.00
100
75
63
48
35
25
20
17
11
CV – 55 kDa
CIII – 48 kDa
Total protein
C
(kDa)
Extract
CTRL
0.01
0.1
1.00
75
63
48
35
25
20
17
11
CV – 55 kDa
CIII – 48 kDa
Extract
CTRL
0.01
0.1
1.00
Total protein

Figure S2. Uncropped images obtained for Total OXPHOS complex V and III protein, using the primary antibody Total OXPHOS Rodent WB Antibody Cocktail (1:500, ab110413, Abcam, Cambridge, UK). Panel A correspond to n=1 and n=2 and respective total protein level. Panel B correspond to n=3 and n=4 and respective total protein level. Panel C correspond to n=5 and n=6 and respective total protein level, in TM3 cells after the exposure to the extract.
