# Supplementary material for: Pro-Oxidant Anthocyanins-Enriched Fraction Inhibits Androgen Synthesis by Transcriptional Repression of Cyp17a1 Through Nr0b2
Source: Antioxidants (Basel). 2026 Apr 23;15(5):530. doi: 10.3390/antiox15050530 (PMC13203438; doi:10.3390/antiox15050530)
Supplement: Supplementary file 1 [file antioxidants-15-00530-s001.zip › Supplementary NMR Table & Materials 2 9_04_26.pdf]

Pro-oxidant anthocyanins enriched fraction inhibits androgen synthesis by transcriptional repression of *Cyp17a1* through *Nr0b2*

Giuseppe T. Patanè [a,b,c], Ruben J. Moreira [c,d], Ana D. Martins [d], Pedro F. Oliveira [d], Stefano Putaggio [a], Davide Barreca [a], Marco G. Alves [*c]

[a] Department of Chemical, Biological, Pharmaceutical and Environmental Sciences, University of Messina, 98166 Messina, Italy

[b] Prof. Antonio Imbesi" Foundation, University of Messina, Messina, 98100, Italy

[c] Institute of Biomedicine, Department of Medical Sciences (iBiMED), University of Aveiro, 3810-193 Aveiro, Portugal

[d] LAQV-REQUIMTE, Department of Chemistry, University of Aveiro, 3810-193 Aveiro, Portugal

*Corresponding Author(s)-Email: Marco G. Alves email: marcoalves@ua.pt telephone: +351967245248

Supplementary Table S2: Assignment of resonances in the ^{1}H-NMR spectra of Leydig cells media.

	Compound	H multiplicity	Chemical shift, δ (ppm)
**1**	2-Hydroxyisobutyrate	Singlet	1.36
**2**	Acetate	Singlet	1.90
**3**	Alanine	Quartet	3.77
**4**	Betaine	Singlet	3.25
**5**	Creatine	Singlet	3.94
**6**	Glucose	Doublet	5.22
**7**	Glutamine	Triplet	3.75
**8**	Glycine	Singlet	3.55
**9**	Isoleucine	Doublet	1.00
**10**	Lactate	Quadruplet	4.12
**11**	Leucine	Doublet	0.94
**12**	Malonate	Singlet	3.12
**13**	P-Creatine	Singlet	3.92
**14**	Pyruvate	Singlet	2.36
**15**	Sarcosine	Singlet	3.60
**16**	Succinate	Singlet	2.39
**17**	T-Methylhistidine	Singlet	7.04
**18**	Threonine	Multiplet	4.25
**19**	Trymethylamine	Singlet	2.88
**20**	Tyrosine	Doublet	7.18
**21**	Valine	Doublet	1.04

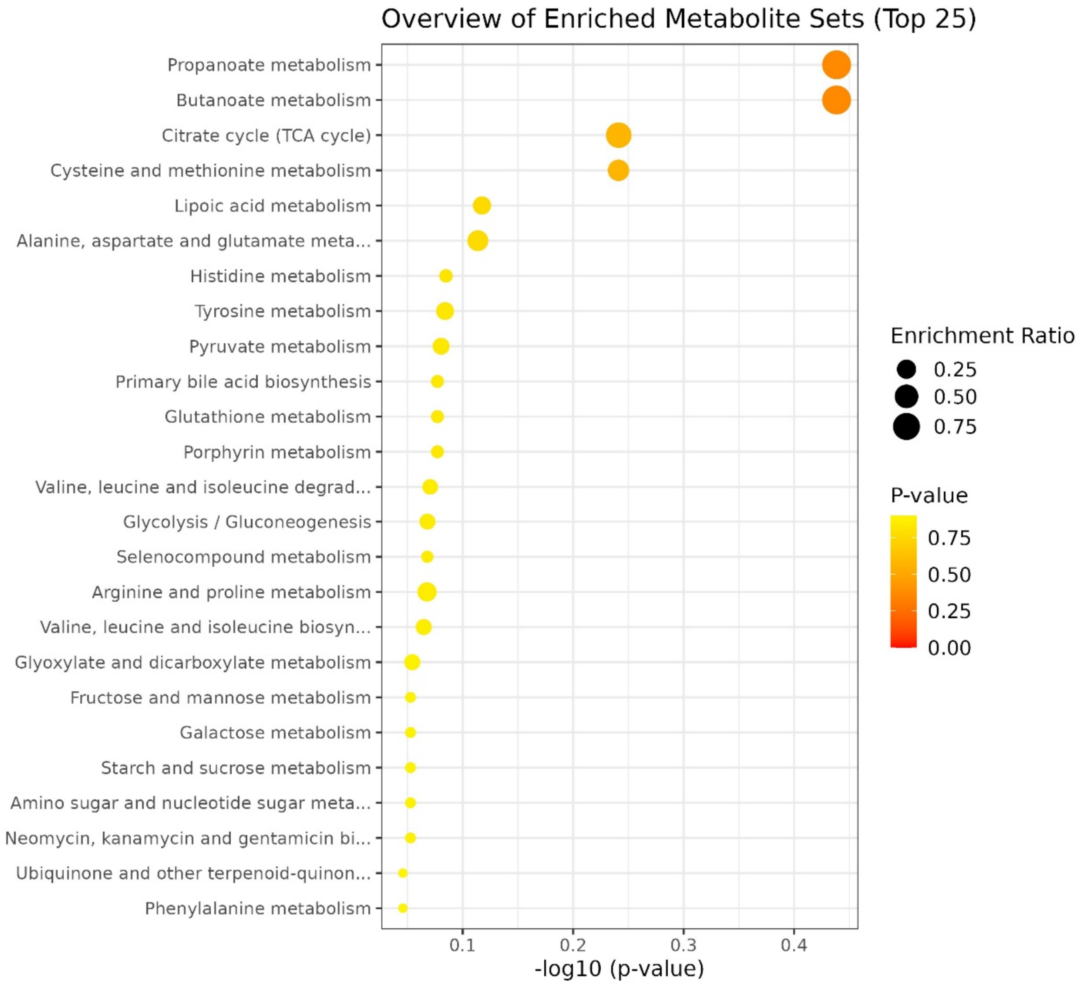

Figure S3. Quantitative enrichment analysis (QEA) of exometabolomic data from control Leydig cells (Group A) and cells exposed to the highest concentration of the anthocyanin-enriched fraction (Group D). The dot plot summarizes the pathway-level results obtained using the QEA module implemented in MetaboAnalyst, based on the quantified extracellular metabolites. Each dot represents a metabolic pathway.
