# Supplementary material for: Pro-Oxidant Anthocyanins-Enriched Fraction Inhibits Androgen Synthesis by Transcriptional Repression of Cyp17a1 Through Nr0b2
Source: Antioxidants (Basel). 2026 Apr 23;15(5):530. doi: 10.3390/antiox15050530 (PMC13203438; doi:10.3390/antiox15050530)
Supplement: Supplementary file 1 [file antioxidants-15-00530-s001.zip › Supplementary Table 1 (genes) 9_04_26.pdf]

Pro-oxidant anthocyanins enriched fraction inhibits androgen synthesis by transcriptional repression of *Cyp17a1* through *Nr0b2*

Giuseppe T. Patanè [a,b,c], Ruben J. Moreira [c,d], Ana D. Martins [d], Pedro F. Oliveira [d], Stefano Putaggio [a], Davide Barreca [a], Marco G. Alves [*c]

[a] Department of Chemical, Biological, Pharmaceutical and Environmental Sciences, University of Messina, 98166 Messina, Italy

[b] Prof. Antonio Imbesi" Foundation, University of Messina, Messina, 98100, Italy

[c] Institute of Biomedicine, Department of Medical Sciences (iBiMED), University of Aveiro, 3810-193 Aveiro, Portugal

[d] LAQV-REQUIMTE, Department of Chemistry, University of Aveiro, 3810-193 Aveiro, Portugal

*Corresponding Author(s)-Email: Marco G. Alves email: marcoalves@ua.pt telephone: +351967245248

Supplementary Table S1: Description of primers for qRT-PCR used in this research.

Gene	Forward primer (5′→3′)	Reverse primer (5′→3′)	AT (°C)
Star	CAGAGGATTGGAAAAGACACGG	AACCTCTGCGCTTGGTACA	58
Cyp17a1	TGTCAGGAAGCCAACTCACT	GCCCAGGACATCCACAATAC	63
Nr0b2	GCACGATCCTCTTCAACCCA	CAGAAGGGTGCCTGGAATGT	58
mtND1	GCATCTTATCCACGCTTCCG	TGGTGGTACTCCCGCTGTAA	58
β2-M	ACGTAACACAGTTCCACCCG	TCTCGATCCCAGTAGACGGT	60

Legend: *Star* – steroidogenic acute regulatory protein; *Cyp17a1*- Cytochrome P450 Family 17 Subfamily A Member 1; Nr0b2 – nuclear receptor subfamily 0 group B member 2; *MT-ND*1 - NADH dehydrogenase 1; β2 M – β2-Microglobulin; AT – annealing temperature.
